# Boost of single-photon emission by perfect coupling of InAs/GaAs quantum dot and micropillar cavity mode

**DOI:** 10.1186/s11671-020-03358-1

**Published:** 2020-07-09

**Authors:** Shulun Li, Yao Chen, Xiangjun Shang, Ying Yu, Jiawei Yang, Junhui Huang, Xiangbin Su, Jiaxin Shen, Baoquan Sun, Haiqiao Ni, Xingliang Su, Kaiyou Wang, Zhichuan Niu

**Affiliations:** 1 State Key Laboratory for Superlattice and Microstructures, Institute of Semiconductors,Chinese Academy of Sciences, Beijing, 100083 China; 2 Center of Materials Science and Optoelectronics Engineering,University of Chinese Academy of Sciences, Beijing, 100049 China; 3 Beijing Academy of Quantum Information Sciences, Beijing, 100193 China; 4 Institute of Photonics and Photonic Technology, Northwest University, Xian, 710127 China; 5 State Key Laboratory of Optoelectronic Materials and Technologies, School of Electronics and Information Technology, Sun Yat-sen University, Guangzhou, 510275 China; 6 School of Microelectronics, Xidian University and The State Key Discipline Laboratory of Wide Band Gap Semiconductor Technology, Xian, 710071 China; 7 Laboratory of Solid Quantum Material Center, College of Physics and Electronic Engineering, Shanxi University, Taiyuan, 030006 China

**Keywords:** Single-photon source, Micropillar, Cavity mode, Weak coupling regime

## Abstract

We proposed a precise calibration process of Al _0.9_*Ga*_0.1_As/GaAs DBR micropillar cavity to match the single InAs/GaAs quantum dot (QD) exciton emission and achieve cavity mode resonance and a great enhancement of QD photoluminescence (PL) intensity. Light-matter interaction of single QD in DBR micropillar cavity (Q ∼ 3800) under weak coupling regime was investigated by temperature-tuned PL spectra; a pronounced enhancement (14.6-fold) of QD exciton emission was observed on resonance. The second-order autocorrelation measurement shows *g*^(2)^(0)=0.070, and the estimated net count rate before the first objective lens reaches 1.6×10^7^ counts/s under continuous wave excitation, indicating highly pure single-photon emission at high count rates.

## Introduction

Quantum light source that emits single photons is the key device of quantum information processing [1–3]. High photon extraction efficiency, strong suppression of multi-photon emission, and high indistinguishability [4] of the emitted single photons are desired. Among all the ways to realize quantum light sources such as atomic systems [5], parametric down-conversion [6], or vacancy centers in diamond [7, 8], semiconductor InAs/GaAs quantum dots (QDs) are promising candidates to realize practical monolithic quantum light sources for quantum communication and other applications such as quantum-enhanced sensing [9] or quantum imaging [10]. The advantages of InAs/GaAs QDs include extremely narrow linewidth [4], stable and on-demand emission with high single photon emission rate (can be enhanced by the cavity coupling) [11], easy to tune through physical multi-fields [12–14], more suitable for fiber-array coupling output [15], and the wavelength is tunable (840 ∼1300 nm at present) for potential telecom quantum information application [16]. Despite its advantages, the key issue to realize a practical QD single-photon source is how to further improve the brightness (i.e., count rates) of single photon source, which will greatly improve the efficiency of quantum information transmission [4]. Therefore, it is necessary to improve the extraction efficiency of QD emission and improve their brightness by means of coupling QDs with microcavities, including micropillars [11], microdisk [17], photonic crystals [18], and microstructures like microlenses [19–22]. Meanwhile, the light-matter interaction of different systems and the coupling effect in the visible and infrared range have been extensively studied [23–27]. In recent years, the study of semiconductor QDs embedded in micropillar cavities and their cavity electrodynamic effects has attracted extensive attention for high *Q* value, low mode volume [11], and its convenience in direct fiber-coupling output [28–33]. Furthermore, a perfect resonant coupling of the cavity mode with QD luminescence wavelength is another key challenge [34, 35]. In this work, a pronounced crossover phenomenon of exciton energy and micropillar cavity mode (Q ∼ 3800) and an enhancement of exciton emission intensity were observed and an experimental precise cavity mode calibration process was proposed, which can achieve a perfect coupling of micropillar cavity mode and wavelength of QDs and then produce a single photon source with high brightness and high single-photon purity.

## Methods

**Fig. 1:**
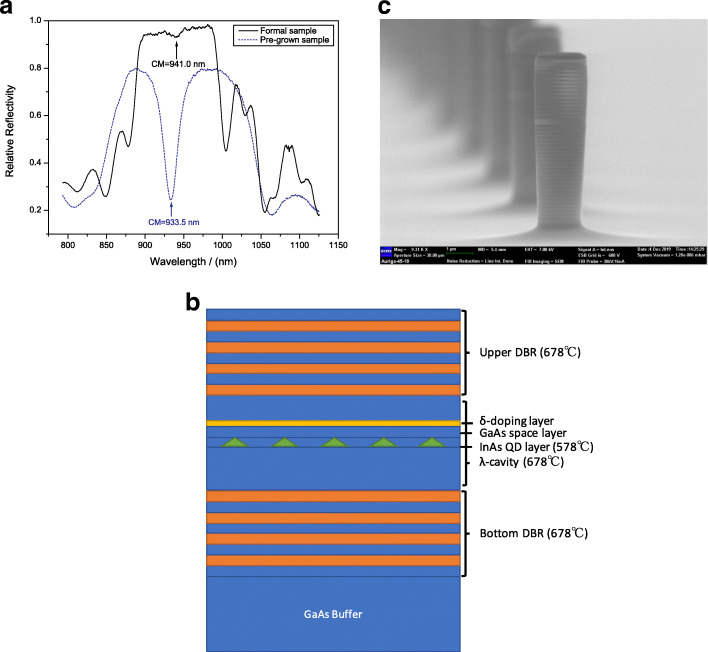
**a** The reflection spectra at room temperature (*T* = 300K) of the pre-grown sample with 6.5 pairs lower and 4 pairs upper DBR and the formal sample after the precise cavity mode calibration process with 25.5 pairs lower and 15 pairs upper DBR. **b** Schematic structures of the formal sample. **c** Scanning electron microscope (SEM) image of the micropillar cavity with diameter of 2.0 *μ*m and height of 6.5 *μ*m

The investigated sample was grown by solid-source MBE (VEECO Gen930 system) on semi-insulating GaAs(001) substrate. The sample structure consists of, in sequence, 500-nm-thick GaAs buffer layer, 25.5 pairs Al _0.9_*Ga*_0.1_As/GaAs bottom DBR, one *λ*-thick GaAs cavity, and 15 pairs Al _0.9_*Ga*_0.1_As/GaAs upper DBR with the same period. In the center of one *λ*-thick GaAs cavity, the active InAs/GaAs QDs layer for single-photon emission was grown in Stranski-Krastanov growth mode with indium deposition amount gradient on chip so that certain regions satisfy the proper deposition amount for dilute single QD formation with exciton emission wavelength around 910 ∼930 nm [36]. The above-lying layer of the InAs QDs layer is a 10-nm-thick GaAs cladding layer. Above the cladding layer is a Be *δ*-doping layer with an average sheet doping density of about 2×10^8^*cm*^−2^ to increase QD brightness [37, 38], and the overall schematic structures of the formal sample was demonstrated in the Fig. 1b.

In order to couple the DBR cavity mode with the emission wavelength of InAs QD perfectly, we performed a precise cavity mode calibration process. The calibration process is as follows: firstly, determine the InAs/GaAs single QD exciton emission wavelength by *μ*PL spectroscopy (usually, ∼ 920 nm at 10 K); then, grow a pre-grown QD sample with a fewer Al _0.9_*Ga*_0.1_As/GaAs DBR periods (6.5 pairs lower and 4 pairs upper DBR) with the thicknesses defined by *λ*/4*n* (*λ*: the designed center wavelength of the DBR cavity, *n*: material refraction index); after growing the pre-grown sample, measure its optical reflection spectra at 300 K and 77 K respectively to obtain the shift rate of the cavity mode; then, define the mismatch ratio of DBR thickness at the same temperature; for here, we have defined the measured cavity mode position of the pre-grown sample (e.g., *λ*1) and the mismatch ratio is *λ*/*λ*1 so that we grow the formal sample (25.5 pairs lower and 15 pairs upper DBR) with DBR thickness (i.e., growth time) multiplying the mismatch ratio. The samples grown by this method can accurately obtain a perfect phase matching in DBR microcavity as designed, thus coupling with the emission wavelength of single InAs QDs and achieving an optimal enhancement of QD emission.

In this work, the micropillar arrays were fabricated on the DBR cavity-coupled QD samples by electron beam photolithography (EBL) and inductive coupled plasma (ICP) etching; the serial number is designed and fabricated on the surface of the sample to identify every single micropillar. In temperature-tuned PL spectra measurements, the sample was cooled in a cryogen-free bath cryostat with the temperature finely tuned from 4 K to 60 K and excited by a He-Ne laser at the wavelength of 632.8 nm. The confocal microscope setup with an objective lens (NA, 0.70) focuses the laser into a spot in a diameter of 2 *μ*m and collects the luminescence effectively into a spectrograph, which enables scanning of micro-region to search single QD exciton spectral lines. Micro-photoluminescence (*μ*PL) spectra was detected by a 0.75-m-long focal length monochromator equipped with a liquid-nitrogen-cooled Si CCD detector for spectrograph. The attenuation slice was set in the spectral system to tune the excitation power, as to identify the style of exciton. To investigate the coupling phenomenon of exciton and cavity mode, the *μ*PL spectra was measured at various stable temperatures ranging from 6 to 45 K. To investigate the radiative lifetime of the exciton, a time-correlated single photon counting (TCSPC) board were used for time-resolved *μ*PL measurement. To measure the second-order autocorrelation function *g*^(2)^(*τ*), the QD spectral line luminescence was sent to a fiber-coupled Hanbury-Brown and Twiss (HBT) setup [20] and detected by two Si avalanched single-photon counting modules (SPCM-AQR-15; time resolution, 350 ps; dark count rate, 80 counts/s; dead time, 45 ns) and a time coincidence counting module.

## Results and discussion

Figure 1a shows the reflection spectra at room temperature (*T* = 300 K) of the pre-grown sample with 6.5 pairs lower and 4 pairs upper DBR and the formal sample after the cavity mode calibration process with 25.5 pairs lower and 15 pairs upper DBR stacks. The cavity mode calibration process is to compare the measured central fundamental cavity mode (933.5 nm of pre-grown sample at 300 K) with the emission wavelength of InAs QD (917.5 nm at 6.0 K), and then convert both into the same temperature to obtain the mismatch ratio. When growing the formal sample, multiply the DBR growth time by the mismatch ratio to achieve accurate calibration of the cavity mode to couple with the emission wavelength of single InAs QDs. Comparing the reflection spectra of the pre-grown sample and the formal sample, cavity mode position was moved from 933.5 to 941.0 nm as expected. Figure 1c shows the scanning electron microscope (SEM) image of the micropillar cavity. As shown in the SEM image, the micropillars with a diameter of 2.0 *μ*m and a height of 6.5 *μ*m have very smooth sidewalls and high-quality structure appearance, and the InAs QDs were embedded in a *λ*-thick GaAs cavity and sandwiched between 25.5 pairs lower and 15 pairs upper DBR stacks in order to enhance the photon collection efficiency.

**Fig. 2:**
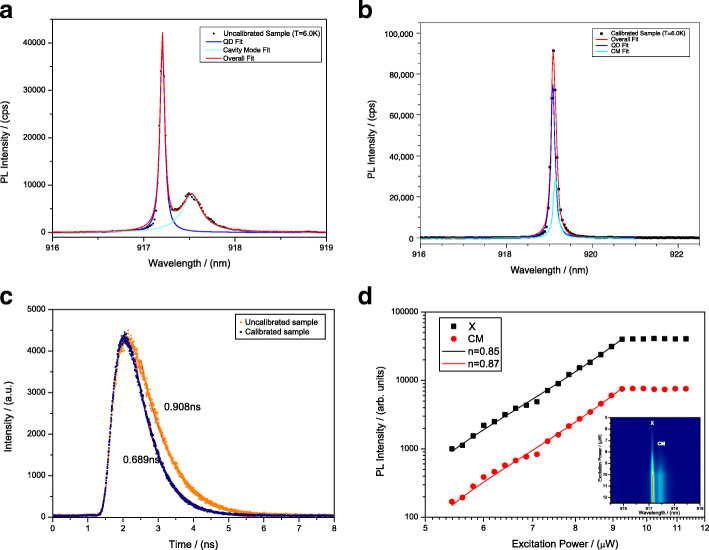
**a***μ*PL spectra of the QD exciton of the uncalibrated sample at 6.0 K with the exciton (X) line and the cavity mode (CM) line. **b***μ*PL spectra of the QD exciton of the calibrated sample at 6.0 K. Colored lines: Lorentz fitting of the experimental data. **c** Time-resolved measurements of uncalibrated sample and the calibrated sample at 6.0 K. **d** Excitation power-dependent *μ*PL spectra of the uncalibrated sample at 6.0 K; inset: integrated PL intensity of X and CM as a function of excitation power in a log-log scale

Figure 2a shows the exciton line (X) at 917.24 nm and cavity mode (CM) line at 917.54 nm which is the typical non-resonance circumstance of the QD embedded in a micropillar cavity. In order to couple the DBR cavity mode with the wavelength of InAs QD perfectly, a precise cavity mode calibration process was carried out. After calibrating, the cavity mode coupled with the QD perfectly, which shows in the Fig. 2b where there is only X line at 919.10 nm. On resonance, compared with non-resonance circumstance, the PL intensity of the X line get enhanced greatly from 42k to 95k cps. The detuning energy of the QD and CM is 73.4 *μeV* based on the fitting results. According to the time-resolved measurements of resonant and non-resonant circumstance, the perfect coupling of QD and the cavity mode reduces the lifetime from 0.908 to 0.689 ns as shown in Fig. 2c. The strong enhancement of emission intensity and the decrease of lifetime are related to the increased spontaneous emission rate for the resonant QD exciton due to the Purcell effect [39].

The excitation power-dependent *μ*PL spectra of InAs/GaAs QD coupled with micropillar was studied by using continuous-wave (CW) He-Ne laser for above-band excitation as Fig. 2d shows. The quality factor (*Q*) of the micropillar cavity is estimated to be 3800. The identification of these emission lines is demonstrated by their power dependencies. With the increase of the excitation power, the PL intensity of the X line and cavity mode line is enhanced obviously. The integrated PL intensity of both X line and CM lines in a log-log scale shows a linear dependence under low excitation power and saturated under high excitation power. The solid lines are linear fitting to the data in a double-logarithmic plot. The fitting results show that the PL intensity and excitation power have an exponential relationship where the *n* (*I*∝*P*^*n*^) of X and CM line are 0.85 and 0.87 respectively, indicating that the emission line type is exciton line. The deviation of the exponent from the ideal value expected for the exciton line (*n*_*X*_=1) might be due to the effect of nonradiative recombination centers in the vicinity of the QDs [4], which affect the carrier distribution at different carrier densities.

**Fig. 3:**
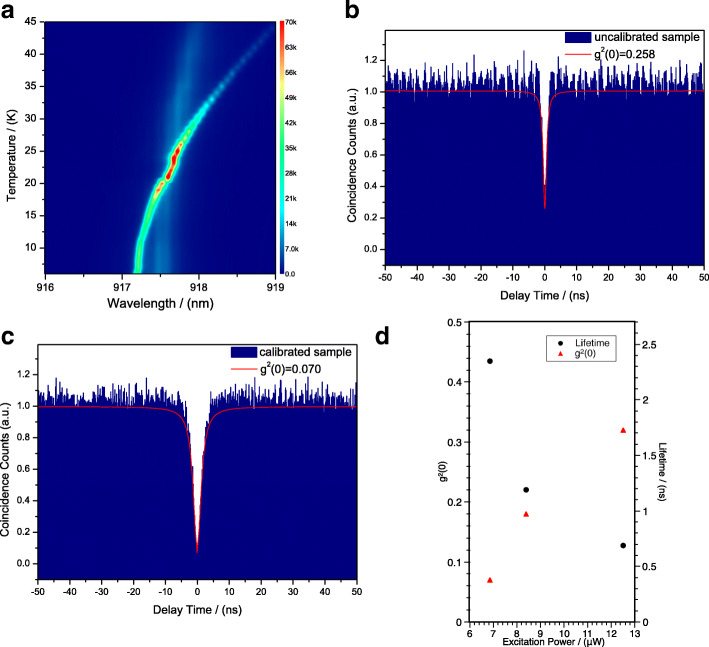
**a** Contour of temperature-tuned PL spectra of the uncalibrated sample from 6.0 to 45.0 K. The second-order correlation function *g*^(2)^(*τ*) of the QD exciton (X) line under CW excitation of the sample without the calibration process (**b**) and the calibrated sample (**c**). **d** The radiative lifetime and *g*^(2)^(0) of the exciton emission for the calibrated sample under different excitation power

Figure 3a shows the temperature-tuned PL spectra of the uncalibrated sample. According to the Fig. 3a, the exciton (X) line and the cavity mode (CM) line moved at different shift rates by increasing the temperature from 6.0 to 45.0 K. The CM line shifted from 917.54 nm (6.0 K) to 918.01nm (45.0 K) and the CM shift rate is 0.018 *μ*eV/K, while the X line shifted from 917.24 nm (6.0 K) to 919.07 nm (45.0 K) and the X shift rate is about 0.069 *μ*eV/K. The exciton emission shift rate is greater than the cavity mode shift rate as expected. By comparing the curves of X and CM lines, the two curves intersect at the temperature of 24.0 K, indicating a point where the exciton and the cavity mode reach resonance at 24.0 K. At resonance, there is an enhancement of the exciton emission and the observed enhancement of emission is about 14.6-fold where the exciton PL peak intensity increased from 6.5×10^3^ cps to 9.5×10^4^ cps. The pronounced crossing phenomenon of the cavity mode and exciton energies is demonstrated in the Fig. 3a, which indicates that the light-matter interaction conforms to weak coupling regime.

To confirm the anti-bunching effect of single-photon emission of the QD exciton line, the second-order correlation function *g*^(2)^(*τ*) of both the uncalibrated sample and the calibrated sample was measured with HBT setup under CW excitation. Figure 3b and c shows the measured second-order correlation function of the X line under resonance as a function of the delay time *τ*. The data could be fitted with the following expression: $g^{(2)}(\tau)=1-[1-g^{(2)}(0)]exp(-\frac {\mid \tau \mid }{T})$ [40]. Figure 3b shows the second-order correlation function of the sample without the calibration process. In order to obtain a better single-photon performance, the single QD exciton X line of the uncalibrated sample was tuned into resonance under 24.0 K to measure the *g*^(2)^(*τ*). The second-order correlation function at zero delay of the uncalibrated sample under temperature-tuned resonance is *g*^(2)^(0)=0.258. Figure 3c shows the *g*^(2)^(*τ*) of the QD exciton after the precise calibration process under 6.0 K, where *g*^(2)^(0)=0.070. Both are less than 0.5, which indicates an obvious anti-bunching effect and proves a single-photon emitter with strong suppression of the multi-photon emission at zero time delay. Due to the precise cavity mode calibration process, the perfect coupling between QD exciton and cavity mode improved the single-photon purity from 74.2% to 93.0%. Figure 3d shows the radiative lifetime and *g*^(2)^(0) of the exciton emission for the calibrated sample under different excitation power. The curves fitting of $g^{(2)}(\tau)=1-exp(-\frac {\mid \tau \mid }{T})$ gives the exciton radiative lifetime (*T*), and the figure demonstrates that *T* becomes shorter as the excitation power increases, while *g*^(2)^(0) at lower excitation power is smaller than that at saturated excitation power, indicating a purer single photon emission under lower excitation power.

To obtain the net single-photon count rate of the QD exciton after the precise calibration process, we estimated all the optical loss including the photon detection efficiency and the transmission loss. The photon detection efficiency of the Si detector is 33%, and the transmission loss is 81% including objective lens collection efficiency (66%), narrow bandpass filter efficiency (40%), fiber collimator (80%), and multimode fiber coupling efficiency (90%). Based on the count rate (1.0×10^6^ counts/s) on two Si single-photon detectors in the coincidence measurements and corrected photon count rate by the factor of [1−*g*^(2)^(0)]^1/2^ [41], we estimate the net single-photon count rate is 1.6×10^7^ counts/s at the first objective lens. The results indicate that during the sample growth stage, the perfect coupling between the cavity mode and QD exciton can produce a purer and brighter single-photon source through the precise calibration process.

## Conclusions

In conclusion, we presented a bright single-photon source at 919 nm by fabricating InAs/GaAs QD in a micropillar Al _0.9_*Ga*_0.1_As/GaAs DBR cavity. The temperature-tuned PL spectra demonstrates a pronounced (14.6-fold) enhancement of QD exciton emission at the crossing with the cavity mode under the weak coupling regime. With the help of the precise cavity mode calibration progress, it is easy to obtain a perfect phase matching in DBR microcavity to reach an optimal cavity mode spacial distribution as theoretically designed and thus achieving an optimal enhancement of QD emission. The perfect coupling between QD exciton and cavity mode enhanced the PL intensity by 2.3 times and the single-photon purity improved from 74.2 to 93.0%. The second-order autocorrelation measurement yielded *g*^(2)^(0)=0.070 under cavity resonance, indicating single-photon emission at a high count rate with 1.6×10^7^ counts/s before the first objective lens. This work demonstrates a highly feasible method for perfect coupling of QD with cavity mode and the fabrication of high-purity and high-brightness single-photon sources.

## Supplementary information

**Additional file 1** See supplementary information for detailed discussion on the evolution of GaAs layer, In(Ga)As wetting layer and QD formation, as well as the variation of the PL spectrum and the AFM images during this process.

## Data Availability

The datasets used and/or analyzed during the current study are available without restriction from the corresponding author on reasonable request.

## Abbreviations

DBR: Distributed Bragg reflector
HBT: Hanbury-Brown and Twiss
ICP: Inductive coupled plasma
MBE: Molecular beam epitaxy
QDs: Quantum dots
SEM: Scanning electron microscope
NA: Numerical aperture
CW: Continuous wave
SPSs: Single-photon sources
CM: Cavity mode
TCSPC: Time-correlated single-photon counting
SPCM: Single-photon counting modules
*μ*PL: Microphotoluminescence.
