# Supplementary material for: Boost of single-photon emission by perfect coupling of InAs/GaAs quantum dot and micropillar cavity mode
Source: Nanoscale Res Lett. 2020 Jul 9;15:145. doi: 10.1186/s11671-020-03358-1 (PMC7347735; doi:10.1186/s11671-020-03358-1)
Supplement: Supplementary file 1 — Additional file 1 See supplementary information for detailed discussion on the evolution of GaAs layer, In(Ga)As wetting layer and QD formation, as well as the variation of the PL spectrum and the AFM images during this process. [file 11671_2020_3358_MOESM1_ESM.pdf]

Supplementary information：Boost of Single-Photon Emission by Perfect Coupling of InAs/GaAs Quantum Dot and Micropillar Cavity Mode

Shulun Li, Yao Chen, Xiangjun Shang, Ying Yu, Jiawei Yang, Junhui Huang, Xiangbin Su, Jiaxin Shen, Baoquan Sun, Haiqiao Ni*, Xingliang Su, Kaiyou Wang and Zhichuan Niu

In this supplementary material, we present the discussion on the evolution of GaAs layer, In(Ga)As wetting layer and QD formation, as well as the variation of the PL spectrum and the AFM images during this process.

The atomic force microscope (AFM) images (figure S1) and μPL spectra (Figure S2) reflect the evolution of QD formation. Before the formation of the wetting layer, the AFM image shows atomic stage (Figure S1.a) without QD and the μPL spectrum (blue line) shows only GaAs peak at 820 nm; when the wetting layer was formed, the AFM image shows circular atomic stage (Figure S1.b) and the μPL spectrum (red and black lines) shows a sharp peak around 867 nm; when dense small QDs (height of 1-5 nm) was formed as the AFM images shows (Figure S1.c), the μPL spectrum shows a broad profile around 875 nm; when single QDs with a height of 6-8 nm was formed among the dense small QDs (Figure S1.d), the μPL spectrum shows sharp and narrow lines around 920 nm; when these 6-8 nm-height QDs become more (Figure S1.e), the μPL spectrum shows a broad profile 980 nm; continue to increase the In deposition amount, these QDs become larger and their density reduces to ~100 μm^{-2} (Figure S1.f), with QD height distributed from 5 nm to 11 nm or higher, corresponding to several peaks in μPL spectra around 1000-1300 nm.

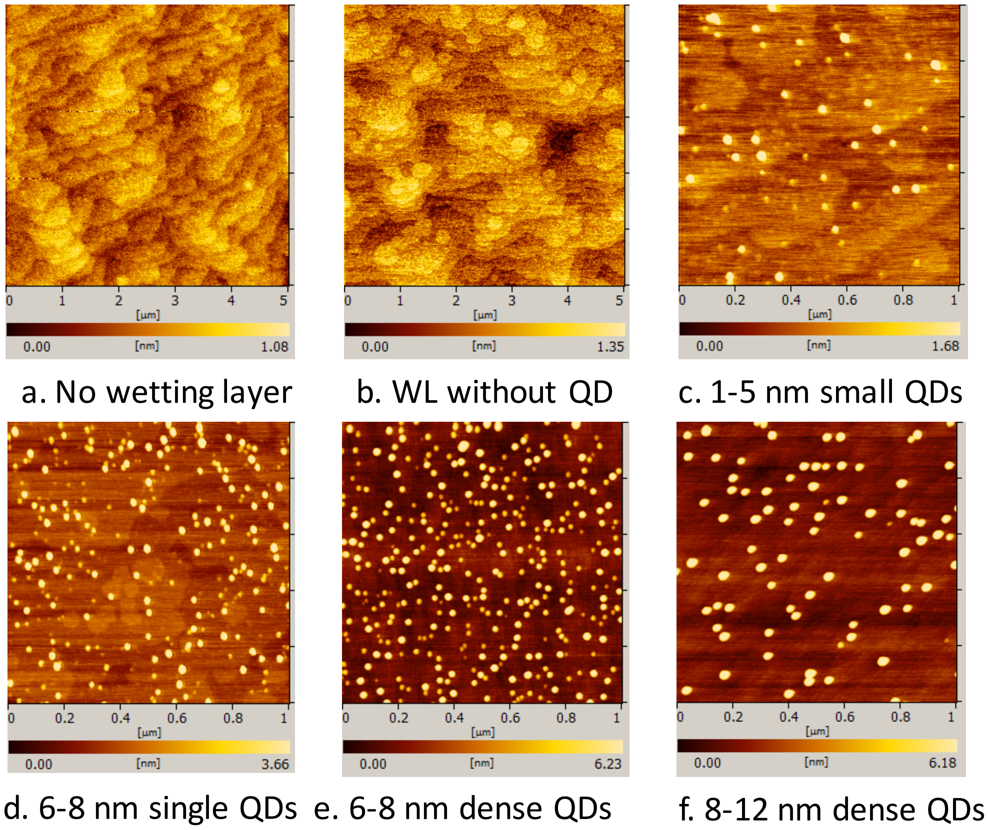

Figure S1 AFM images in the process of the evolution of WL to InAs QDs

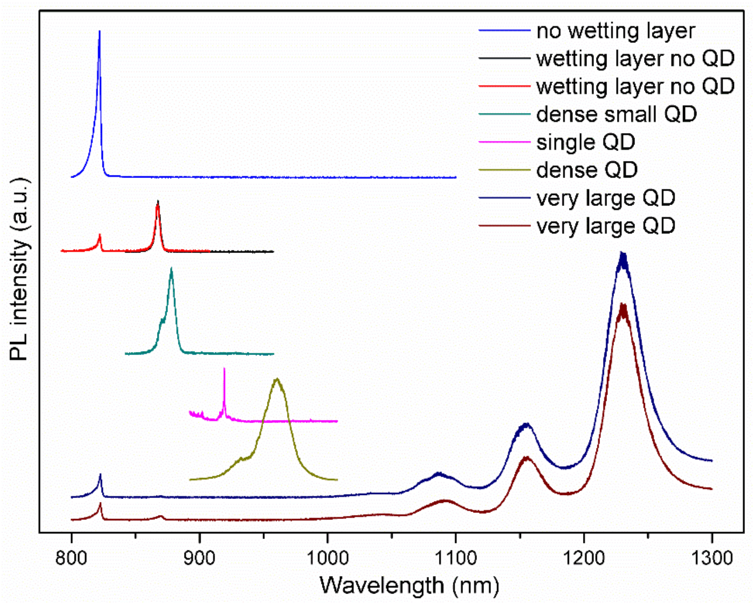

Figure S2 μPL spectra in the process of the evolution of WL to InAs QDs

Figure S3 shows the AFM images of InAs QDs on different regions of the uncapped pre-grown sample with the same structure. On the top of the pre-

grown sample, InAs QD was grown again by the same parameters for AFM measurements. As the figure S3.a shows, the small quantum dots with a height of 1~5nm usually formed on a low Indium deposition area, which spectrally corresponds to a wide profile at 860~880 nm. The dilute single InAs QDs with a height of 6~8 nm (indicated by the black arrow in figure S3.b) among the previous 1~5 nm-height small QDs are formed by Indium immigration, these dilute single InAs QDs exhibit sharp spectral lines at wavelength of 910~930 nm, corresponding to exciton emission in these single QDs (detailed in figure S2). In a region with a more Indium deposition amount on the wafer, larger QDs with a height of 8~12 nm (figure S3.c) are formed and show a broad spectral profile around 1000-1300 nm (see also figure S2).

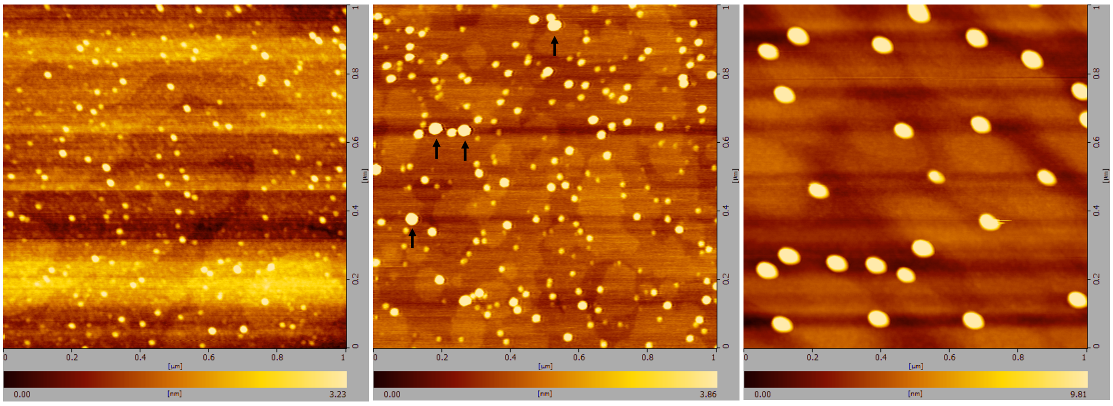

a b c

Figure S3 AFM images of uncapped InAs QDs on the pre-grown sample

To confirm the emission characteristics of the pre-grown sample, we performed PL spectroscopy on our pre-grown sample with only 5~6 pairs of the top DBRs (i.e. a weak DBR planar cavity with a small Q) that can extract emission in a broad spectral range, rather than the formal DBR pillar sample (top DBR: 16

pairs) that only extract a narrow width (~0.5 nm) spectrum. As figure S4 in supplementary documents shows, there are multiple luminescence peaks of different exciton states between 910~920 nm. The different exciton states were identified through the excitation power dependences. From the identification of the featured multi-exciton emission spectral lines in a QD, distinguished from the wetting layer emission (only one PL spectral peak around 867 nm in linewidth of 10 nm as the above shown), single QD formation is proved. The formal sample discussed in the manuscript has a very narrow (<0.5 nm) cavity mode due to more pairs of DBR, and only one exciton state can be coupled and filtered out. This is why there is only one emission line in the PL spectrum of the formal sample.

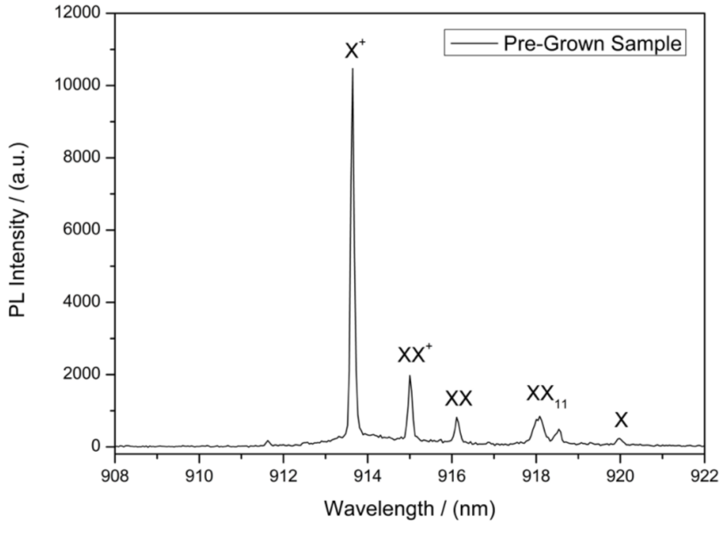

Figure S4 μPL spectrum of the pre-grown sample with multi-exciton emission lines.
